# Topical cannabidiol (CBD) in skin pathology – A comprehensive review and prospects for new therapeutic opportunities

**DOI:** 10.4102/safp.v64i1.5493

**Published:** 2022-05-30

**Authors:** Lehlohonolo Makhakhe

**Affiliations:** 1 Department of Dermatology, Faculty of Health Science, University of the Free State, Bloemfontein, South Africa; 2 The South African Institute of Dermatology, Bloemfontein, South Africa

**Keywords:** cannabis, dermatology, new modality of treatment, anti-inflammatory, topical usage

## Abstract

Humans have utilised cannabis products in various forms throughout the recorded history. To date, more than 500 biologically active components have been identified in the plants of the *Cannabis* genus, amongst which more than 100 were classified as phytocannabinoids (exocannabinoids). The plant genus *Cannabis* is a member of the plant family Cannabaceae, and there are three primary cannabis species which vary in their biochemical constituents: *Cannabis sativa, Cannabis indica* and *Cannabis ruderalis*. There has been a growing level of interest in research on the topical usage of a cannabis-based extract as a safer and more effective alternative to the usage of topical corticosteroids in treating some dermatoses. Together with the discovery of the cannabinoid receptors on the skin, it has been further illustrated that topical cannabis has anti-inflammatory, anti-itching, analgesics, wound healing and anti-proliferative effects on the skin.

## Introduction

Cannabinoids work on the endocannabinoid system (ECS), which consists of a series of neuromodulatory lipids and receptors located throughout the brain and the central and peripheral nervous system, which accept endogenous cannabinoids (anandamide, 2-arachidonoylglycerol) and phytocannabinoids (plant-based).^1^

The (cannabinoid-1) CB_1_ and (cannabinoid-2) CB_2_ receptors are two main receptors within the ECS. The two most well-researched phytocannabinoids are the delta-9 tetrahydrocannabinol (THC) and cannabidiol (CBD). Tetrahydrocannabinol is the primary psychoactive component of cannabis which is responsible for the ‘high’ associated with cannabis use.^2^

In general, cannabis that has high levels of the psychoactive cannabinoid, delta9-tetrahydrocannabinol (9-THC), and low levels of the non/antipsychoactive cannabinoid, CBD, is referred to as ‘marijuana’. Cannabis that has high levels of CBD, and very low insignificant levels of 9-THC, is referred to as ‘industrial hemp’, or ‘hemp’, and has no psychoactive effects.^3,4^

Elements of the ECS were extensively documented in epidermal keratinocytes, melanocytes, mast cells and cutaneous immune regulatory system. Moreover, cannabinoids were shown to suppress *in vitro* proliferation (and differentiation) of cultured epidermal keratinocytes, alter pain and stimulate wound healing, and have anti-microbial and pruritic properties.^5,6^

Substances penetrate the outer layer of the skin and permeate from one layer to another. Substances are transferred through the main barrier of the skin, the stratum corneum, by passive diffusion into the underlying viable layers of the epidermis.^7^

Uptake of a substance is proportional to compound’s concentration gradient between the formulation and the base of the stratum corneum. The extent of the uptake varies widely with the physicochemical properties of the xenobiotic, the formulation and application of the product, and with skin conditions.^7,8^

There is always a need to seek more clinically efficient, safer and widely available methods of treating medical conditions, skin pathology is no different. Even more interesting, topical use of cannabis as a form of treatment covers a wider scope of skin disorders as highlighted in the discussion.

The purpose of this review article is to look broadly at the medicinal benefits of cannabis in treating a variety of skin disorders. It is a succinct summary of current knowledge based on a meta-analysis of global literature on the subject matter. As such, no article was excluded when literature was gathered; this was with a deliberate aim of excluding bias, promoting a fair, balanced and comprehensive evidence-based article.

## Discussion

**FIGURE 1:**
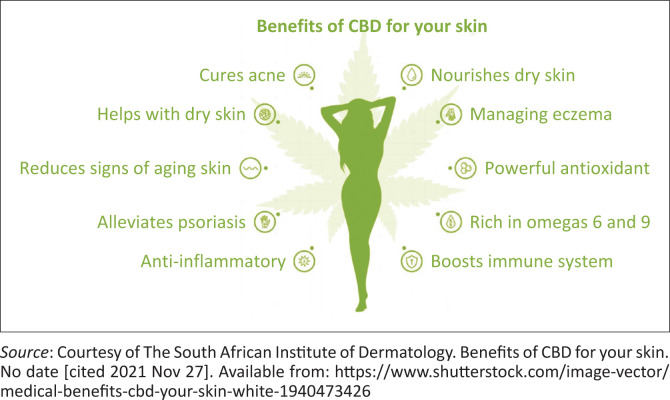
Benefits of cannabidiol for your skin.^9^

Cannabinol-based studies promise to be a key feature in literature and might lead to a breakthrough that will add a different dimension to how we see skin pathology treatment for a plethora of skin diseases (see Figure 1).

## Sebaceous gland related disorders

### Acne and rosacea

Acne is a common skin disease characterised by elevated sebum production and inflammation of the sebaceous glands. It has been previously shown that non-psychotropic phytocannabinoids exert complex anti-acne effects by normalising ‘pro-acne agents’-induced excessive sebaceous lipid production, reducing proliferation and alleviating inflammation in human sebocytes. Based on their remarkable anti-inflammatory actions, phytocannabinoids could be efficient, yet safe novel tools in the management of cutaneous inflammations.^10^

*Sebaceous glands* are holocrine glands found over the entire surface of the body except the palms, soles and dorsum of the feet. They are the largest and mostly concentrated in the face and scalp, where they are the sites of the origin of acne. The normal function of sebaceous glands is to produce and secrete sebum. The sebaceous glands further transport antioxidants in and on the skin, and exhibit a natural light protective activity. They possess an innate antibacterial activity and have a pro- and anti-inflammatory function.^11^

It has been clinically observed that cannabinoid abuse can be accompanied by acne. This already highlights how cannabinoid signalling may influence human sebocyte biology. Indeed, expression of CB_1_ (in the differentiated, central cells) and CB_2_ (predominantly in the basal, non-differentiated sebocytes) receptors has been well-documented in human skin.

In a small, single-blind, split-face study, a cream containing 3% *Cannabis* seed extract was applied twice daily to the cheeks of patients for 12 weeks. The treatment was found to be efficient in reducing sebum production and erythema compared to the vehicle treated side.^6^

## Hair growth disorders

### Alopecia, hirsutism, hypertrichosis

Alopecia is defined as loss of hair, whilst hirsutism is excessive hair growth in women at sites where hair is under androgenic control. Hypertrichosis is defined as excessive hair growth in both density and length beyond the accepted limits for age.^12^

Considering the well-known anti-inflammatory and immunosuppressive effects of cannabinoids, it is not surprising that certain data suggest involvement of cannabinoid dysregulation in the development of alopecia areata. CB_1_ receptor agonists have been found to be of significant benefit in treating hirsutism and hypertrichosis. In contrast, CB_1_ receptor agonists have been found to treat alopecia areata.^5^

## Skin tumours

### Basal cell carcinoma, squamous cell carcinoma and melanoma

Non-melanoma skin cancers (specifically basal cell carcinoma [BCC] and squamous cell carcinoma [SCC]) are the most common malignancies in humans. Given the growth inhibitory effects of cannabinoids on the main receptors, studies of anti-tumour therapy have been and continues to be extensively studied. Through the induction of apoptosis, inhibiting tumour angiogenesis and halting the cell cycle, cannabinol extracts have been found to be beneficial in treating non-melanoma skin cancers.^13^

Cannabinoids have been found to also have a therapeutic effect on melanomas. This is believed to be through arresting cell cycles, apoptosis and other less-known mechanisms.^14^

## Melanocytes and pigmentation disorders

### Vitiligo and melasma

It has been well-documented that a functional ECS is present in primary human melanocytes, the respective target receptors (CB_1_, CB_2_ and the transient receptor potential vanilloid channel [TRPV-1]) and their metabolic enzymes. Higher concentrations of some endocanabinnoids induce normal human epidermal melanocyte apoptosis through a TRPV1-mediated pathway that increases deoxyribonucleic acid (DNA) fragmentation and p53 expression. However, at lower concentrations, endocannabinoids and other CB_1_-binding endocannabinoids dose-dependently stimulate melanin synthesis and enhance tyrosinase gene expression and activity. Endocannabinoids seem to have a double effect on human melanocytes: (1) to induce melanogenesis at low concentrations through CB_1_ receptors (vitiligo and other depigmentation skin disorders) and (2) to induce apoptosis at high concentrations through TRPV1 receptors in treating melanomas and melasma.^5,15,16,17^

## Wound healing properties

Considering that cannabinoid signalling regulates fibroblast functions, proliferation and differentiation of epidermal keratinocytes, as well as cutaneous inflammation, it is not surprising that it also influences the complex process of cutaneous wound healing.

Murine data obtained after skin incision suggested that the expression pattern of CB_1_ and CB_2_ can be characterised by dynamic alterations during wound healing in various immune cells as well as in fibroblasts/myofibroblasts. Besides this, several additional lines of evidence support the concept that CB_1_, and especially CB_2,_ can influence wound healing.^5,18^

## Cutaneous inflammation and immunity

### Dermatitis

Studies have demonstrated that topical application of the cannabinoids receptor-1-specific agonist (CB1R) significantly accelerates recovery of the epidermal barrier function in acutely abrogated skin. Anti-inflammatory activities in both the acute irritation and the chronic irritation application were observed, which are consistent with past study results. Whilst further investigations should be performed to clarify the potential involvement of other signalling pathways, current results suggest that topical CB1R agonist treatment can be a potential therapeutic option for acute and chronic inflammatory skin diseases, including atopic dermatitis, contact dermatitis and psoriasis.^19^

## Epidermal proliferation

### Psoriasis vulgaris

Cannabinoids are known to have anti-inflammatory and immunomodulatory properties. Cannabinoid receptor 2 is expressed mainly on leukocytes and is the receptor implicated in mediating many of the effects of cannabinoids on immune processes. Agonists were shown to act directly on T-cells, as exposure of cluster of differentiation 3+ (CD3+) cells to these compounds completely inhibited their action in a reconstituted mixed lymphocyte reaction. Because the pathogenesis of psoriasis largely involves T-cell lymphocyte mediated immune properties and keratinocyte proliferation, cannabinoids are viewed as a subject of great interest in future modalities of treatment.^20,21,22^

## Topical anti-itching properties

Pruritus is an uncomfortable localised or widespread sensation that triggers the intense urge to scratch. It is a common symptom in dermatologic conditions and is a dermatologic manifestation of systemic diseases. A topical endocanabinnoids-containing emollient provided an average of an 86% reduction in subjective pruritus. In a 3-week open-label study involving 21 patients with uremic pruritus, a topical application of endocanabinnoids-containing cream twice a day for three weeks resulted in total elimination of pruritus in more than 38% of the subjects, and another 52% of them reported significantly reduced pruritus. Because no control group was included in these studies, it is unclear how much improvement in pruritus would be obtained by applying the emollient vehicle alone. Nevertheless, topical preparations containing endocannabinoids may provide relief from pruritus by downregulating mast cell degranulation, inhibiting inflammatory cytokines, decreasing the tumour necrosis factor > (TNF->) during inflammation or providing an analgesic effect on their own.^23,24,25^

## Topical analgesic properties

Studies have shown that endogenous cannabinoids (endocannabinoids) as well as synthetic cannabinoid agonists have antinociceptive and anti-inflammatory effects.^24,25,26,27^

## Skin barrier repairing properties, dry skin and permeability enhancement properties

The stratum corneum lipids, composed predominantly of ceramides, free fatty acids and cholesterol, are synthesised and stored in lamellar granules, and as precursors extruded into the extracellular space between the stratum corneum and stratum granulosum. A lack of or imbalance in these lipids can lead to inadequate repair of the skin, skin dryness and permeability challenges.^27^

Studies have demonstrated that the absence of CB_1_ delayed permeability barrier recovery, whilst it was accelerated by a lack of CB_2_. It was further found that in line with these observations, lamellar body secretion as well as the expression of certain late differentiation marker proteins such as filaggrin, loricrin and involucrin as also affected by the endocanabinnoid system. This suggests that using cannabinoids antagonists may play a key role in skin barrier repair properties.^5,23^

## Anti-microbial effects

Cannabis sativa has long been known to contain antibacterial cannabinoids, whose potential to address antibiotic resistance has not yet been investigated. All five major cannabinoids (CBD, cannabichromene, cannabigerol, 9-tetrahydrocannabinol and cannabinol) showed potent activity against a variety of methicillin-resistant Staphylococcus aureus (MRSA) strains of current clinical relevance.^6^

## Conclusion

Of the five major cannabinoid groups, a special focus is on CBD, which continues to be extensively studied through ongoing phase 1 clinical trials, looking at appropriate dosages for different conditions, frequency of applications, best formulation for application, that is, cream, ointment, paste, and so forth as a vehicle in delivering the active components.

In most reviewed literature, there remain limited details on acute topical side effects and long-term efficacy of this specific mode of treatment. Ongoing research must then be promoted, focusing on chances of clinical relapses with discontinued usage, clinical tolerance, association between dosages and skin irritability, possibility of synergy when combining different groups of cannabinoids and placing level of evidence of topical cannabinoids usage in treating every identified skin pathology.

However, there is overwhelming clinical evidence suggesting the beneficial effects of topical cannabinoids in treating a myriad of skin conditions. There is still much research that needs to be conducted, carrying the potential to revolutionise and widen the scope of dermatology treatment modalities.
